# A Druggable FOXA1-Glucocorticoid Receptor Transcriptional Axis Drives Tumor Growth in a Subset of Non-Small Cell Lung Cancer

**DOI:** 10.1158/2767-9764.CRC-23-0310

**Published:** 2023-09-07

**Authors:** M. Dorso, Payal T. Patel, Aleksandr Pankov, Jacob A. Boyer, Rajesh K. Soni, Isabella S. Del Priore, Omar Hayatt, Amanda Kulick, Connor J. Hagen, Elisa de Stanchina, Melissa R. Junttila, Anneleen Daemen, Lori S. Friedman, Ronald C. Hendrickson, Sarat Chandarlapaty

**Affiliations:** 1 Human Oncology and Pathogenesis Program, Memorial Sloan Kettering Cancer Center, New York, New York.; 2 Pharmacology Graduate Program, Weill Cornell Medicine, New York, New York.; 3 ORIC Pharmaceuticals, South San Francisco, California.; 4 Gerstner Sloan Kettering Graduate Program, Sloan Kettering Institute, New York, New York.; 5 Microchemistry and Proteomics Core, Sloan Kettering Institute, New York, New York.; 6 Antitumor Assessment Core, Memorial Sloan Kettering Cancer Center, New York, New York.; 7 Breast Medicine Service, Department of Medicine, Memorial Sloan Kettering Cancer Center, New York, New York.

## Abstract

The FOXA1 pioneer factor is an essential mediator of steroid receptor function in multiple hormone-dependent cancers, including breast and prostate cancers, enabling nuclear receptors such as estrogen receptor (ER) and androgen receptor (AR) to activate lineage-specific growth programs. FOXA1 is also highly expressed in non–small cell lung cancer (NSCLC), but whether and how it regulates tumor growth in this context is not known. Analyzing data from loss-of-function screens, we identified a subset of NSCLC tumor lines where proliferation is FOXA1 dependent. Using rapid immunoprecipitation and mass spectrometry of endogenous protein, we identified chromatin-localized interactions between FOXA1 and glucocorticoid receptor (GR) in these tumor cells. Knockdown of GR inhibited proliferation of FOXA1-dependent, but not FOXA1-independent NSCLC cells. In these FOXA1-dependent models, FOXA1 and GR cooperate to regulate gene targets involved in EGF signaling and G_1_–S cell-cycle progression. To investigate the therapeutic potential for targeting this complex, we examined the effects of highly selective inhibitors of the GR ligand-binding pocket and found that GR antagonism with ORIC-101 suppressed FOXA1/GR target expression, activation of EGF signaling, entry into the S-phase, and attendant proliferation *in vitro* and *in vivo*. Taken together, our findings point to a subset of NSCLCs harboring a dependence on the FOXA1/GR growth program and provide rationale for its therapeutic targeting.

**Significance:**

NSCLC is the leading cause of cancer deaths worldwide. There is a need to identify novel druggable dependencies. We identify a subset of NSCLCs dependent on FOXA1-GR and sensitive to GR antagonism.

## Introduction

Inhibitors of lineage-specific master regulatory transcription factor (TF) complexes are some of the most successful approaches in oncology practice. Among the most well-established instances of successful targeting of TF complexes in cancer have been antagonists of nuclear receptor function (1). Nuclear hormone receptors are lineage-defining oncogenic drivers in luminal-type breast and prostate cancer. Luminal-type breast cancers, which make up approximately 70% of all breast cancers, are defined by the expression of and dependence upon the estrogen receptor (ER; ref. 2) and nearly 90% of primary prostate cancers are defined by expression and dependence on the androgen receptor (AR; ref. 3). In keeping with these dependencies, drugs targeting AR and ER are widely used and highly effective (4–6).

One requirement for oncogenic nuclear receptors to perform master regulatory functions is a permissive chromatin landscape at corresponding target genes. The pioneer factor FOXA1 is a critical mediator in this regard (7–11). Binding of FOXA1 to target gene enhancers and recruitment of chromatin remodeling enzymes primes the genomic environment and is required for the transcriptional response to ligand stimulation of the nuclear receptor (12, 13). As a result of the dynamic cooperation between FOXA1 and nuclear receptor function, loss of FOXA1 expression is sufficient to restrict ER and AR transcriptional activity, cell proliferation, and tumor growth in ER^+^ breast cancers and AR^+^ prostate cancers, respectively (13, 14). Therefore, tumor expression of FOXA1 in ER^+^ breast and AR^+^ prostate cancers is often a predictive indicator of sensitivity to nuclear hormone receptor–targeted therapies (15–18).

Although the oncogenic functions of FOXA1 have been extensively studied in breast and prostate cancer, its potential role in regulating cancer-specific transcriptional programs in other tumor lineages is just beginning to be appreciated. Enhanced FOXA1 expression has recently been shown to be involved in the metastatic transition of pancreatic ductal adenocarcinomas (19). Other groups have reported increased FOXA1 mRNA expression in colorectal tumors (20), anaplastic thyroid carcinoma (21), and esophageal and lung adenocarcinomas (LUAD; ref. 22) pointing to a potential oncogenic role for FOXA1 in these tumor lineages. In this study, we investigated the essentiality of FOXA1 across cell line lineages and identified a subset of non–small cell lung cancer (NSCLC) cell lines that is FOXA1 dependent. Endogenous immunopurification of FOXA1 in dependent NSCLC models revealed that FOXA1 forms a complex with the glucocorticoid receptor (GR) on DNA. We further demonstrated that a subset of FOXA1-dependent NSCLC models is also dependent on GR, and that FOXA1 and GR converge on the regulation of targets involved in the activation of growth factor signaling and cell-cycle progression. Finally, we showed that pharmacologic antagonism of GR can recapitulate the effects of FOXA1 and GR knockdown and have profound antitumor effects in this subset of NSCLC. Collectively, our results provide a plausible basis for exploring GR antagonism as a potential therapeutic approach for NSCLC.

## Materials and Methods

### Plasmids

SGEP (pRRL), LT3GEPIR, and LT3REVIN were gifts from Charles Sawyers. The psPAX2 and pVSVG vectors were kind gifts from Ping Chi. Short hairpin RNA (shRNA) sequences were designed by the Zuber Lab and are as follows: shRenilla – TGCTGTTGACAGTGAGCGCAGGAATTATAATGCTTATCTATAGTGAAGCCACAGAT GTATAGATAAGCATTATAATTCCTATGCCTACTGCCTCGGA; shFOXA1#1 – TGCTGTTGACAGTGAGCGAAAAGACAATACTGCTGTTATATAGTGAAGCCACAGATGTATATAACAGCAGTATTGTCTTTCTGCCTACTGCCTCGGA; shFOXA1#2 – TGCTGTTGACAGTGAGCGACAGGATGTTAGGAACTGTGAATAGTGAAGCCACAGATGTATTCACAGTTCCTAACATCCTGGTGCCTACTGCCTCGGA; shGR#1 – TGCTGTTGACAGTGAGCGAAAGCTGTAAAGTTTTCTTCAATAGTGAAGCCACAGATGTATTGAAGAAAACTTTACAGCTTCTGCCTACTGCCTCGGA; shGR#2 – TGCTGTTGACAGTGAGCGCTCAGACCTGTTGATAGATGAATAGTGAAGCCACAGATGTATTCATCTATCAACAGGTCTGATTGCCTACTGCCTCGGA. The pBIND-GR/pGL4.35 two-vector luciferase assay system was purchased from Promega (REF# E1581/E1370).

### Cell Lines

All cell lines were maintained at 37°C and 5% CO_2_ in a humidified atmosphere. The NCI-H441, NCI-H1437, NCI-H2122, and NCI-H2009 cell lines were obtained from the ATCC. The HCC-44 cells were obtained from DMSZ. NCI-H3255 cells were gifted by Ingo Mellinghoff. A549 cells were provided by Charles Rudin. PC-9 cells were a gift from Neal Rosen. NCI-H2122 and NCI-H358 were gifted by Piro Lito. NCI-H441, NCI-H1437, NCI-H2122, HCC-44, NCI-H3255, A549, NCI-H358, and PC-9 cells were grown in RPMI medium supplemented with 10% FBS. NCI-H2009 cells were grown in DMEM-F12 supplemented with 5% heat-inactivated FBS, 0.005 mg/mL insulin, 0.01 mg/mL transferrin, 30 mmol/L sodium selenite (final concentration), and 10 nmol/L beta-estradiol (final concentration). All the cell culture media were supplemented with 100 μg/mL penicillin, 100 μg/mL streptomycin, and 4.5 mmol/L glutamine. All cell lines were authenticated by short tandem repeat genotyping and tested negative for routinely tested for *Mycoplasma* contamination. All cell lines were cultured and collected within 25 passages.

### Drugs and Reagents

Dexamethasone (MK-125) was purchased from Selleck Chemicals. ORIC-101 and OP-4954 were obtained from ORIC Pharmaceuticals. All the drugs were dissolved in DMSO.

### Lentiviral Infections

HEK293CT packaging cells were plated at 2.5  ×  10^7^ cells/ tissue culture dish (10 cm in diameter) and transfected with 4.5 μg of the SGEP lentiviral vector (encoding shRenilla, shFOXA1 #1, shFOXA1 #2, shGR #1, and shGR #2), 4.5 μg of psPAX2, and 1 μg of pVSVG with X-tremeGENE HP(Roche) according to the manufacturer's protocol. For experiments utilizing inducible shRNA expression vectors, 4.5 μg of the LT3GEPIR (shRenilla, shFOXA1 #1, and shFOXA1 #2) were cotransfected with psPAX2 and pVSVG. Conditioned medium containing recombinant lentivirus was collected and filtered through non-pyrogenic filters with a pore size of 0.45 μm (Millipore). Samples of these supernatants were applied immediately to target cells, which had been plated 18 hours before infection, at a density of 3  ×  10^6^ cells/tissue culture dish (10 cm in diameter). Polybrene (Sigma) was added at a final concentration of 8 μg/mL, and the supernatants were incubated with the cells for 12 hours. After infection, the cells were placed in fresh growth medium and cultured as described previously. Selection with 1 μg/mL puromycin was initiated 48 hours after infection. To generate double knockdown cells, NCI-H441 cells infected with LT3GEPIR shRenilla or shFOXA1 #1 were infected with LT3REVIN shFOXA1 #1 or shGR#1 virus and selected with 1 μg/mL puromycin and 500 μg/mL neomycin 48 hours after infection.

### Proliferation Assays

Approximately 500–1,000 cells in 200 μL of media were seeded per well of a 96-well plate with six replicates per sample. If necessary, the cells were treated the following day (day 0). On the days when the plates were measured, 25 μL of resazurin (R&D Systems AR002) was added per well and incubated for 4 hours at 37°C. Plates were then read using SpectraMax M5 (Molecular Devices) and the results were analyzed using Softmax Pro 6.2.2 software with an Endpoint Readtype (Excitation: 560 nmol/L, Emission: 590 nmol/L). The results were first normalized to blank media with no cells and then to the fluorescence reading at day 0 to determine relative cell proliferation.

### Immunoblotting

The cells were washed once with cold PBS and scraped off the plate with a rubber policeman. The cell suspension was briefly centrifuged to pellet the cells, PBS was removed, and the cell pellet was stored at −80 °C until lysis. For cell lysis, the pellets were resuspended in RIPA buffer (Pierce) supplemented with protease and phosphatase inhibitors (Pierce). Lysates were cleared by centrifugation at 16,000 × *g* for 10 minutes, and the protein concentration was determined using the BCA kit (Pierce), which measures the reduction of Cu2+ to Cu1+ by protein in an alkaline medium. For each sample, 25 μg of protein lysate was loaded onto 4%–12% SDS-PAGE gels (Invitrogen) for electrophoresis and immunoblotting against FOXA1 (Abcam #23738), GAPDH (Cell Signaling Technology #5174), Actin (Cell Signaling Technology #4970), GR (Cell Signaling Technology #12041), Cyclin D1 (Cell Signaling Technology #55506), phospho-RB (S807/811; Cell Signaling Technology #8516), Lamin B2 (Cell Signaling Technology #12255), EGFR (Cell Signaling Technology #4267), phospho-EGFR Y1068 (Cell Signaling Technology #3777), phospho-AKT S473 (Cell Signaling Technology #4060), phospho-4E-BP1 S65 (Cell Signaling Technology #13443), phospho-S6 S235/236 (Cell Signaling Technology #4858), phospho-MEK1/2 Ser221 (Cell Signaling Technology #2338), phospho-ERK1/2 T202/Y204 (Cell Signaling Technology #4377), and Cleaved PARP Asp214 (Cell Signaling Technology #5625).

### Co-immunoprecipitation Followed by Immunoblotting

Cell pellets were collected as described for immunoblotting. For cell lysis, pellets were resuspended in 1% NP-40 buffer supplemented with protease and phosphatase inhibitors, lysates were cleared, and protein concentration was determined as described for immunoblotting. A total of 5 μg of antibody suitable for immunoprecipitation (IP; Cell Signaling Technology GR mouse mAb #47411 and normal mouse IgG1 sc-3877) was added to 500 μg protein lysate and incubated overnight with rotation at 4°C. A total of 50 μL of precleared protein G beads (Thermo Fisher Scientific catalog no.10003D) were added to the lysate with antibody and incubated with rotation for 3 hours at 4°C. Beads were then isolated using a magnetic rack and washed four times with 1% NP-40 for 5 minutes at 4°C, and then eluted from beads with 4X LDS sample buffer at 70°C for 10 minutes. Eluted immunoprecipitated proteins and input samples were loaded onto SDS-PAGE gels and detected by immunoblotting as described above.

### Chromatin Immunoprecipitation Following by Sequencing

For chromatin immunoprecipitation sequencing (ChIP-seq), H441 cells cultured in full serum-containing media were treated with 100 nmol/L dexamethasone or vehicle (DMSO) for 30 minutes prior to cross-linking and collection. ChIP was performed using the SimpleChIP Enzymatic Chromatin IP kit (Cell Signaling Technology #9003) according to the manufacturer's protocol. Briefly, 4.0 × 10^6^ H441 cells were used per chromatin IP, which was equivalent to 10 μg of chromatin. IPs were performed using 2–10 μg of the following antibodies: GR (Cell Signaling Technology#12041), FOXA1 (Cell Signaling Technology #53528), Normal Rabbit IgG (Cell Signaling Technology #2729). Chromatin preparations were shipped to Genewiz (Azenta) for library preparation and sequencing. ChIP-seq alignments from Genewiz were used to call peaks with MACS, combining both replicates and using a *P* value of 0.001. Peaks that overlap the blacklist file from the Kundaje lab (http://mitra.stanford.edu/kundaje/akundaje/release/blacklists/hg38-human/) were removed. A peak was associated with a gene if the peak was located within the gene body or up to 10 kb upstream or downstream of any isoform from that gene. A gene was defined as shared between GR and FOXA1 if that gene had an associated peak in both the GR and FOXA1 ChIP-seq data. The number of reads overlapping each base were scaled by the total number of reads in that sample and multiplied by 10^6^ to calculate the counts per million (CPM). The ChIP-seq peaks were visually confirmed at genes of interest by visualizing the CPM of each sample within the Integrative Genomics Viewer (IGV).

### Chromatin IP Followed by qPCR

For ChIP-qPCR, chromatin was prepared as described under Chromatin IP followed by sequencing. Following IP, immunoprecipitated chromatin was isolated following SimpleChIP Enzymatic Chromatin IP kit (Cell Signaling Technology #9003) according to the manufacturer's protocol. For ChIP-qPCR analysis, reactions were performed using SimpleChIP Universal qPCR Mastermix (Cell Signaling Technology #88989) according to the manufacturer's protocol. ChIP primer sequences were derived from FOXA1/GR binding sites identified by ChIP-seq and are as follows:

*CCND1* – F: 5′-GACAGAATCCAGCCAGGAGCAG-3′; R: 5′-AGGCAGCCTGCAAATTATTCTCT-3′
*TGFA* – F: 5′-GAATCACCAACAGGCTCTACCAG-3′; R: 5′-TCAAGTGTAACCCTTTCCAGAGAC-3′

### qRT-PCR

RNA was extracted from the cells using the RNeasy Mini Kit (Qiagen) according to the manufacturer's protocol. cDNA was synthesized from 2 μg of RNA from each sample using qScript cDNA SuperMix (Quanta Biosciences) according to the manufacturer's protocol. A total of 2 μL of synthesized cDNA were added to TaqMan PCR Master Mix (Applied Biosystems) along with primers. Relative quantification of each mRNA was performed using the comparative CT method with a ViiA 7 Real-Time PCR system (Applied Biosystems). Samples were run in triplicate and mRNA levels were normalized to those of RPLP0 for each reaction. TaqMan primers were all purchased from Thermo Fisher Scientific. The catalog numbers are as follows:

*RPLP0* (Hs99999902_m1)
*FOXA1* (Hs04187555_m1)
*SGK1* (Hs00178612_m1)
*DUSP1* (Hs00610256_g1)
*FKBP5* (Hs01561006_m1)
*NR3C1* (Hs00353740_m1)
*CCND1* (Hs00765553_m1)
*TGFA* (Hs00608187_m1)

### Rapid Immunoprecipitation and Mass Spectrometry of Endogenous proteins

Rapid immunoprecipitation and mass spectrometry of endogenous protein (RIME) was performed as described previously (23), with an additional disuccinimidyl glutamate cross-linking step prior to formaldehyde cross-linking to strengthen protein–protein interactions. Briefly, 60 million cells per cell line were used for each experiment. A total of 10 μg of antibodies against FOXA1 (Abcam, ab5089), GR (Cell Signaling Technology, 12041), or IgG control (Goat IgG Isotype Control Invitrogen #02-6202) were used for each replicate. Three replicates were performed for each cell line. Immunoprecipitated samples were washed as described previously, digested overnight with trypsin, desalted using C18 zip tips, and then dried by vacuum centrifugation. Each sample was reconstituted in 10 μL 0.1% (v/v) formic acid and 4 μL was analyzed by microcapillary LC/MS-MS using a NanoAcquity (Waters) with a 100-μm inner diameter  ×  10-cm length C18 column (1.7 μm BEH130, Waters) configured with a 180-μm   ×  2-cm trap column coupled to a Q-Exactive Plus mass spectrometer (Thermo Fisher Scientific). The peptides were eluted with a linear gradient of 0%–35% acetonitrile (0.1% formic acid) in water (0.1% formic acid) for 150 minutes at a flow rate of 300 nL/minute. QE Plus was operated in automatic, data-dependent MS-MS acquisition mode with one MS full scan (380–1,800 m/z) at 70,000 mass resolution and up to 10 concurrent MS-MS scans for the 10 most intense peaks selected from each survey scan. Survey scans were acquired in profile mode, and MS-MS scans were acquired in centroid mode at 17,500 resolution and isolation window of 1.5 amu and an isolation energy of 27. The AGC was set to 1  ×  10^6^ for MS1 and 5  ×  10^4^ and 100 ms IT for MS2. Charge exclusion of unassigned and greater than six enabled a dynamic exclusion of 15 seconds.

### RNA sequencing

RNA was extracted from cells using the RNeasy Mini Kit (Qiagen) according to the manufacturer's protocol, with additional incubation with DNase during the wash steps. The frozen total RNA was shipped to Genewiz (Azenta). Sample quality control, library preparation, sequencing, and differential expression analyses were performed by Genewiz.

### Cell-cycle Analysis

For each replicate, 100,000 asynchronous cells per replicate were plated in a 6 cm dish in full serum-containing media. Cells were collected by trypsinization and fixed with 70% ethanol on day 0 or after treatment at the indicated times following treatment with vehicle (DMSO), 10 μmol/L ORIC-101 or 10 μmol/L OP-4954. Following overnight fixation of all samples at −20°C, ethanol was decanted and cells were washed with 1X PBS and then FACS buffer. Following washing, cell pellets were resuspended in either FACS buffer (unstained control) or FACS buffer containing RNase A and propidium iodide. DNA content was analyzed using flow cytometry.

### pBIND-GR Luciferase Assay

293T cells cultured in charcoal-stripped serum (CSS)-containing media were transfected with the pBIND-GR and pGL4.35 plasmids according to the manufacturer's protocol, and treated the following day with DMSO, 100 nmol/L dexamethasone, or 100 nmol/L dexamethasone plus the indicated concentrations of either ORIC-101 or OP-4954 for 24 hours. Luciferase activity was detected using the Dual-Glo Luciferase Assay System (Promega), according to the manufacturer's protocol.

### Animal Studies

NOD.Cg-*Prkdc^scid^ Il2rg^tm1Wjl^*/SzJ (NSG) mice were obtained from Jackson Laboratory. Each mouse was subcutaneously injected with 8 million H441 cells. Once tumor volumes reached an average of 175 mm^3^, mice were treated on a 5 days on/2 days off schedule for 25–35 days with ORIC-101 (75 or 150 mg/kg once daily, orally), or vehicle control, and tumor volumes were recorded every 3–4 days. ORIC-101 was formulated in 0.25% carboxymethyl cellulose (CMC), 0.2% Tween 80, and 5% DMSO. Mice were euthanized if the tumors reached 1,000 mm^3^ or at the end of the experiment. These studies were approved by the Institutional Animal Care and Use Committee Review Board at the Memorial Sloan Kettering Cancer Center.

### Data Availability

The ChIP-seq data supporting this publication have been deposited in NCBI's Gene Expression Omnibus (GEO) and is accessible through GEO Series accession number GSE203036 (https://www.ncbi.nlm.nih.gov/geo/query/acc.cgi?acc=GSE203036).

## Results

### FOXA1 Knockdown Suppresses Growth in a Subset of NSCLC Cell Lines

**FIGURE 1:**
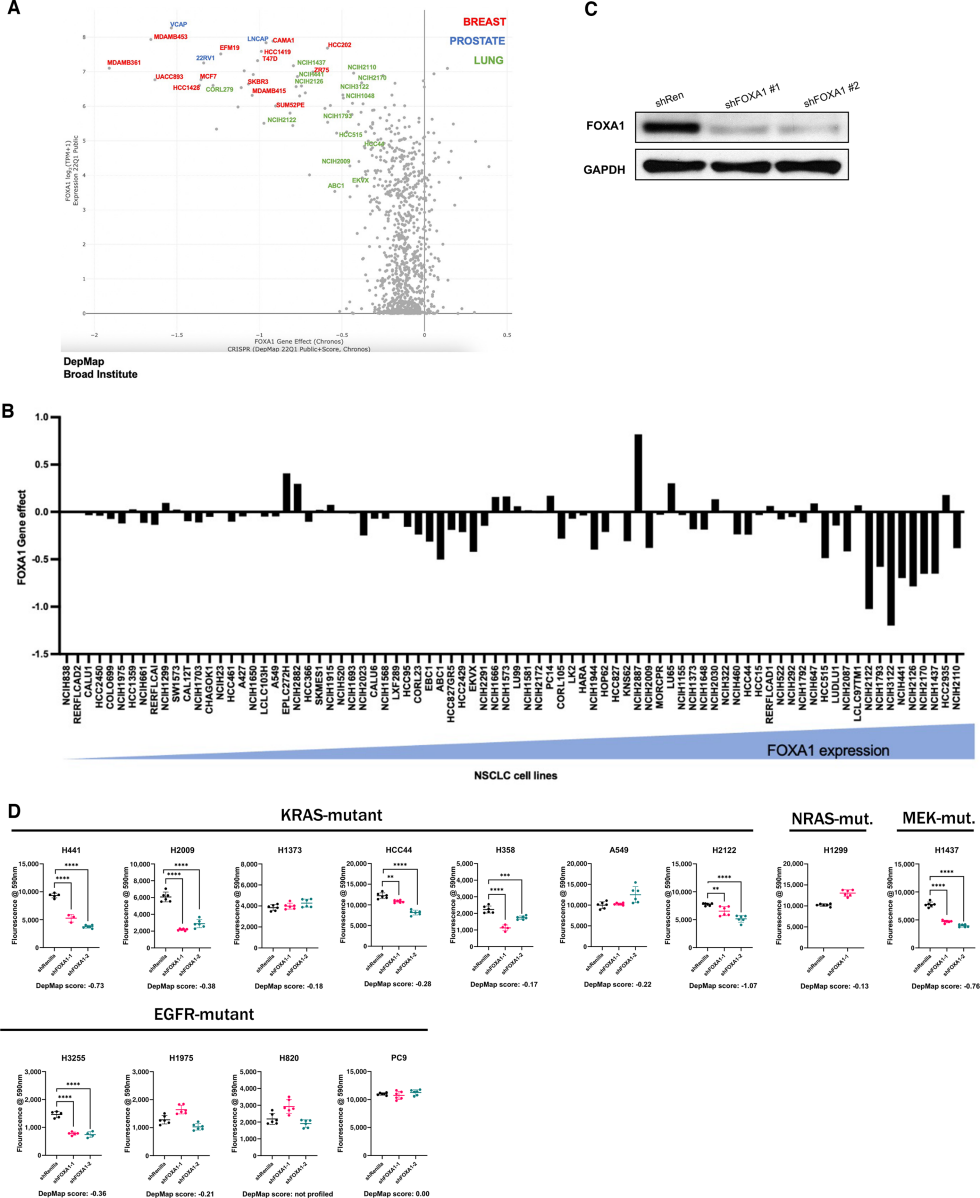
A subset of NSCLC cell lines is FOXA1 dependent. **A,** Scatterplot depicting *FOXA1* gene effect, or degree of essentiality, as a function of its expression. Each dot represents a cell line. Data are curated from The Cancer Dependency Map (Broad Institute). Red text, breast cancer; blue text, prostate cancer; green text, lung cancer cell lines. **B,** Bar graph depicting *FOXA1* gene effect in NSCLC cell lines as in A arranged in order of increasing *FOXA1* expression. Positive gene effect values signify a proliferative advantage conferred by *FOXA1* knockout, whereas negative gene effect values signify a proliferative disadvantage conferred by *FOXA1* knockout. **C,** Immunoblot demonstrating knockdown of FOXA1 in *Renilla* and FOXA1 knockdown in NCI-H441 cells. **D,** Effect of FOXA1 knockdown by shRNA on cell proliferation in NSCLC. Datapoints are means ± SD, *n* = 6. Measurements are fluorescence of Resazurin at 590 nm taken on day 7 after plating. *P* value calculated by Student two-tailed *t* test (*, *P* < 0.05; **, *P* < 0.01; ***, *P* < 0.001; ****, *P* < 0.0001).

FOXA1 is necessary for the viability of breast and prostate cancer cells; however, its role in other cancers is unclear. We examined publicly available genome-wide loss-of-function screening data from the Broad Institute Cancer Dependency Map (DepMap; refs. 24–26) and compared *FOXA1* essentiality as a function of its expression across a broad range of cancer cell lines. As expected, prostate and breast cancer cell lines exhibited the highest degree of FOXA1 dependence, but a cohort of NSCLC cell lines also demonstrated reduced cellular fitness (negative “gene effect” value) following FOXA1 knockout (Fig. 1A; Supplementary Table S1). When we compared the expression of FOXA1 with gene effect (i.e., degree of essentiality/single-guide RNA dropout) across NSCLC cell lines profiled in DepMap, we found that cell lines with higher *FOXA1* expression typically exhibited greater FOXA1 dependence (*P* = 1.6e-4, *n* = 82, DepMap; Fig. 1B). To corroborate these observations, we examined the effects of FOXA1 knockdown on cell proliferation. We infected a panel of 13 NSCLC cell lines with variable FOXA1 dependence with either a shRNA against *FOXA1* (shFOXA1) or a control hairpin targeting *Renilla* luciferase (shRenilla). We found that FOXA1 knockdown suppressed proliferation (30%–70% growth inhibition) in seven out of 13 cell lines assayed (Fig. 1C and D) largely corresponding with the essentiality scores from DepMap. These data revealed a subset of NSCLC cell lines are FOXA1 dependent for growth.

### RIME Analysis of FOXA1-associated Proteins Reveals Interaction with GR in NSCLC Cells

**FIGURE 2:**
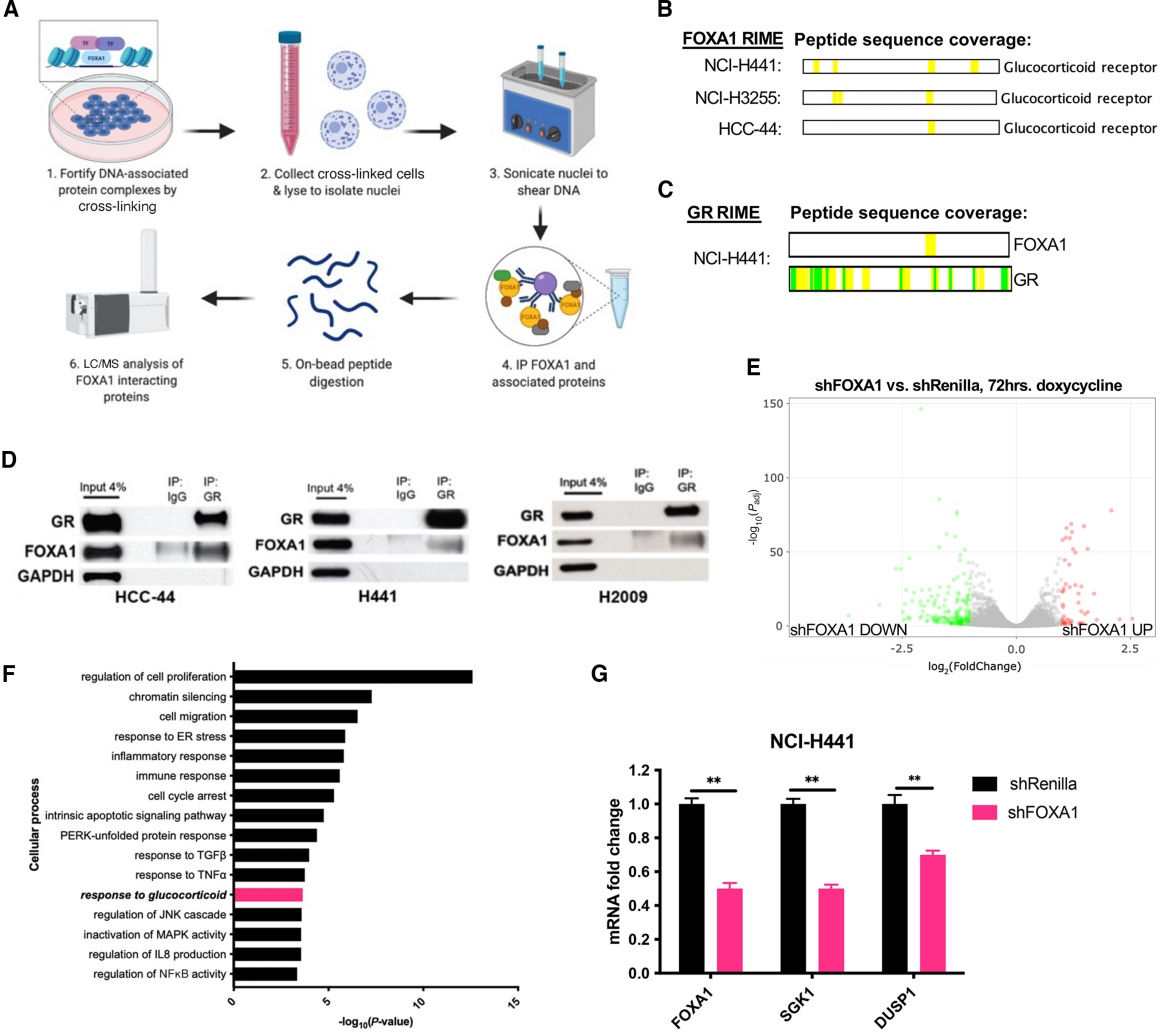
Identification of a FOXA1–GR interaction through endogenous purification of FOXA1-associated proteins using RIME. **A,** Schematic overview of the RIME assay. **B,** Graphic representation of GR protein sequence coverage of unique peptides identified by LC/MS-MS in H441, HCC-44, and H3255 FOXA1 RIME samples. Yellow regions of string represent alignment of peptide sequences to the Mascot database sequences for GR protein. Full list found in Supplementary Table S2. **C,** Graphic representation of FOXA1 and GR protein sequence coverage as in B) in H441 GR RIME samples. **D,** Co-IP of FOXA1 and GR in FOXA1-dependent NSCLC cell lines. GR was immunoprecipitated in full serum-containing media and GR, FOXA1, and GAPDH were immunoblotted in the whole-cell lysate (input), IgG IP, and GR IP fractions. **E,** Volcano plot displaying significantly upregulated (red) or downregulated (green) genes in H2009 doxycycline-inducible shFOXA1 cells compared with shRenilla control cells following 72 hours of 1 μg/mL doxycycline treatment. **F,** Gene ontology pathway analysis of significant differentially regulated genes between H2009 shFOXA1 cells and H2009 shRenilla control cells. **G,** qPCR analysis of fold change in *FOXA1*, *SGK1,* and *DUSP1* mRNA levels in H441 shFOXA1 cells normalized to H441 shRenilla cells treated with 1 μg/mL doxycycline for 48 hours. Values correspond to the mean of three replicates ± SEM. *P* value calculated by Student two-tailed *t* test (*P* < 0.01).

As FOXA1 is known to cooperate with cell type–specific TFs such as ER to mediate its oncogenic effects, we investigated what proteins interacted with FOXA1 in the FOXA1-dependent NSCLC models (27–29). To specifically identify interactions that might transpire on chromatin, we performed RIME (23) in NCI-H441, NCI-H3255, NCI-H2009, HCC-44 (FOXA1-dependent), and NCI-H1373 (FOXA1-independent) cell lines. Three replicates of FOXA1 RIME were conducted for each cell line along with an isotype IgG control (Fig. 2A). We first excluded non-specific proteins found in both FOXA1 RIME and IgG RIME samples, and then narrowed the candidate list to potential functional interactors by focusing on TFs (Supplementary Fig. S1A; Supplementary Table S2) and chromatin remodelers (Supplementary Fig. S1B). Proteins present in at least three out of four FOXA1-dependent cell lines, but not the FOXA1-independent cell line, were ranked by the Mascot ion score, a measure of how closely experimental peptide masses match a database sequence for a given protein (30). Six TFs identified in the FOXA1 interactome fit these criteria: GR, BCL6, STAT3, JUNB, RUNX1, and JUN. Given the cooperativity between FOXA1 and nuclear receptors in breast and prostate cancers, it was notable that the related nuclear receptor, GR, was the top ranked TF among the FOXA1 interactomes in FOXA1-dependent NSCLC (Fig. 2B; Supplementary Fig. S1A). In support of a functional interaction with GR, several known nuclear receptor coregulators also immunoprecipitated with FOXA1, including SWI/SNF complex members, KMT2C, HDAC1/3, NCOA6, and NCOR1/2 (refs. 27, 31–36; Supplementary Fig. S1B).

Although we speculate that FOXA1 may regulate several important cellular processes in NSCLC cells independent of its interaction with GR, we elected to follow-up on this interaction given GR's known role in regulating key transcriptional programs relevant to cancer growth, progression, and therapeutic resistance (37–40) and the potential for therapeutic exploitation of GR. We validated a physical FOXA1–GR interaction by reverse GR RIME in NCI-H441, where FOXA1 was identified in the GR interactome (Fig. 2C), and by co-IP of GR followed by immunoblotting (Fig. 2D). We also detected an interaction between FOXA1 and GR by co-IP in NCI-H2009 cells, where an interaction was not initially detected by RIME.

A functional relationship between FOXA1 and GR was further demonstrated by the transcriptomic effects of FOXA1 knockdown in FOXA1-dependent NSCLC cell lines. Using doxycycline-inducible NCI-H2009 models, we compared the genes and cellular processes altered in shFOXA1 and shRenilla control cells (Fig. 2E). Notably, we observed that the “response to glucocorticoid” gene signature was among the top downregulated pathways by Gene Ontology analysis in FOXA1 knockdown cells compared to the control, in addition to GR-regulated processes including inflammation and immune response (Fig. 2F). We validated differential gene expression data using qRT-PCR for two canonical GR target genes, *SGK1* (41) and *DUSP1* (42), and found that knockdown of FOXA1 significantly reduced the expression of these genes by 30%–50% (Fig. 2G). Taken together, these data reveal that FOXA1 and GR interact within a complex in FOXA1-dependent NSCLC cells and that FOXA1 may be required for the GR transcriptional program.

We reasoned that if FOXA1-dependence was mediated through the transcriptional activity of GR, then FOXA1-dependent cells should also depend on the expression of GR. Therefore, to establish the functional importance of GR in FOXA1-dependent NSCLC, we examined the effect of GR knockdown or knockout on cell proliferation in NCI-H441, NCI-H2009, and NCI-H3255. These FOXA1-dependent cells were infected with lentivirus carrying either GR or *Renilla* luciferase targeting shRNAs. We found that knockdown (Supplementary Fig. S1C and S1D) of GR suppressed the proliferation in each of the three FOXA1-dependent models tested. In contrast, GR knockdown did not suppress proliferation in the FOXA1-independent cell lines PC-9 and A549 (Supplementary Fig. S1E and S1F). These data suggest that FOXA1 and GR may cooperate to promote growth of a subset of NSCLC.

### FOXA1 and GR Regulate the Expression of Targets Involved in Growth Factor Receptor Activation and Cell-cycle Progression

**FIGURE 3:**
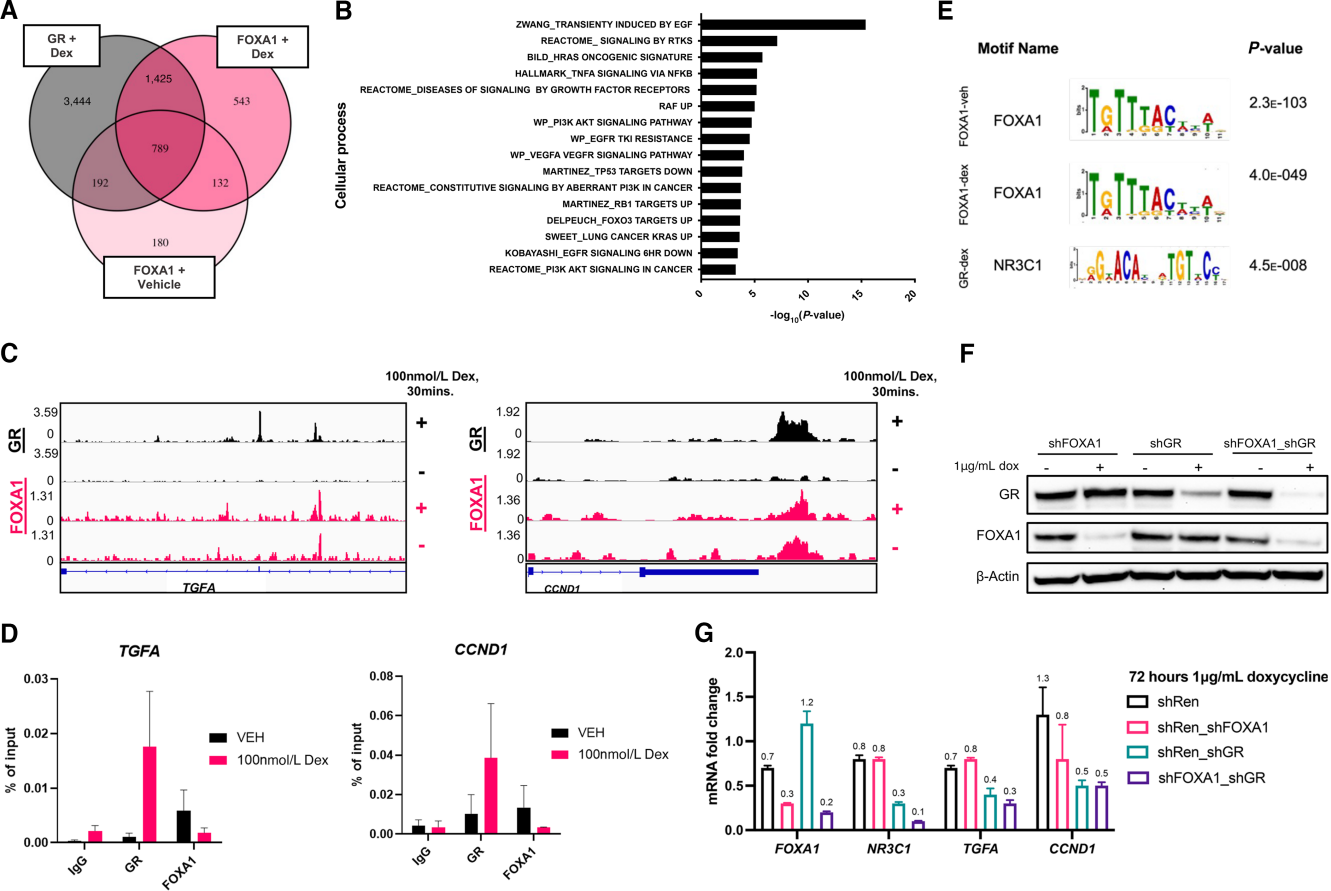
FOXA1 and GR regulate the expression of targets involved in growth factor receptor activation and cell-cycle progression. **A,** Venn diagram depicting overlap in FOXA1 binding events following vehicle or dexamethasone stimulation and GR binding events following dexamethasone stimulation in H441 cells. **B,** GSEA of 789 genes bearing FOXA1 and GR overlapping binding events. **C,** IGV browser views for GR ChIP-seq (black) and FOXA1 ChIP-seq (pink) in H441 cells cultured in full serum media and treated with vehicle or 100 nmol/L dexamethasone for 30 minutes as indicated. Tracks shown are combined peaks from two independent replicates. **D,** ChIP-qPCR analysis of GR, FOXA1, and IgG following 30 minutes of indicated treatment. Rabbit isotype IgG was used as a negative control. Values correspond to mean percentage of input chromatin ± SEM of duplicate qPCR reactions. **E,** Motif analysis at FOXA1 and GR binding regions in indicated treatment conditions. **F,** Immunoblots of FOXA1, GR, and β-Actin in H441 cells expressing inducible shRNAs targeting FOXA1, GR, or FOXA1 and GR, treated with 1 μg/mL doxycycline for 48 hours. **G,** mRNA levels of *FOXA1*, *NR3C1* (GR), and FOXA1/GR targets following treatment with 1 μg/mL doxycycline for 72 hours. Fold change in mRNA is normalized to 0 hour. CT values for each cell line. Values correspond to mean of three replicates ± SD.

To identify the transcriptional targets and cellular programs that may be regulated by the FOXA1–GR complex, we performed ChIP-seq of FOXA1 and GR in NCI-H441 cells and mapped the overlapping binding regions of these two factors. Dexamethasone, a potent GR agonist, was used to induce GR chromatin binding. We integrated GR binding regions identified in the dexamethasone-stimulated cells with FOXA1 binding regions identified in the vehicle- and dexamethasone-stimulated conditions and considered regions of FOXA1/GR binding overlap to include loci where peaks were called for both FOXA1 and GR within 10 kb of a gene. We found a total of 789 gene targets where FOXA1 is bound in both dexamethasone- and vehicle-treated conditions, and GR is subsequently recruited upon dexamethasone stimulation, representing sites where FOXA1 may serve as a licensing factor for GR transcription activity (Fig. 3A; Supplementary Table S3). Through gene set enrichment analysis (GSEA), we determined that overlapping targets were enriched for genes implicated in growth factor receptor and downstream secondary messenger signaling (Fig. 3B). Among these target genes were two well-established regulators of cell proliferation, *TGFA,* which encodes TGFα, an activating ligand for EGFR, and *CCND1*, which encodes cyclin D1 (Fig. 3C and D) and promotes G_1_ checkpoint kinase activity (43, 44). We performed motif analysis on these sites and validated that Forkhead box and nuclear receptor motifs were enriched (Fig. 3E). To determine whether the expression of these targets was regulated by FOXA1 and GR, we measured the mRNA levels of these transcripts in doxycycline-inducible H441 shRenilla, shFOXA1, shGR cells, or a model expressing shRNAs targeting both FOXA1 and GR (shFOXA1_shGR) treated with doxycycline to induce knockdown for 72 hours. Knockdown of FOXA1 alone led to a modest downregulation of *TGFA* and *CCND1.* However, we observed marked inhibition of target expression in shGR or shFOXA1_shGR cells compared with control shRenilla cells (Fig. 3F and G).

### Growth Promoting Functions of GR are Uncoupled from Corticosteroid-activated functions of GR in FOXA1/GR-dependent NSCLC

Nuclear receptors can be activated in a ligand-dependent or -independent manner. Therefore, we considered the possibility that ligand activation of GR drives the activation of oncogenic transcription programs and cell proliferation in FOXA1/GR-dependent NSCLCs. To assess the contribution of GR transactivation to cell proliferation, we cultured two FOXA1/GR-dependent NSCLC models, NCI-H441 and NCI-H3255, in media containing either hormone replete (FBS) or hormone-deprived (CSS) serum and stimulated these cells with either vehicle or dexamethasone. While complete hormone deprivation robustly inhibited growth in both models, readdition of dexamethasone did not restore the proliferative rate, nor did it enhance the proliferation of cells cultured in hormone-replete conditions (Supplementary Fig. S2A and S2B). In line with this observation, dexamethasone treatment did not induce expression of FOXA1/GR target genes whose expression was inhibited upon FOXA1 or GR knockdown (TGFA and CCND1), but induced the expression of canonical, dexamethasone-induced genes such as SGK1, DUSP1, and FKBP5 (ref. 45; Supplementary Fig. S2C). These data suggest that a subset of the growth-promoting functions of GR is independent of the ligand-activated transcription program.

**FIGURE 4:**
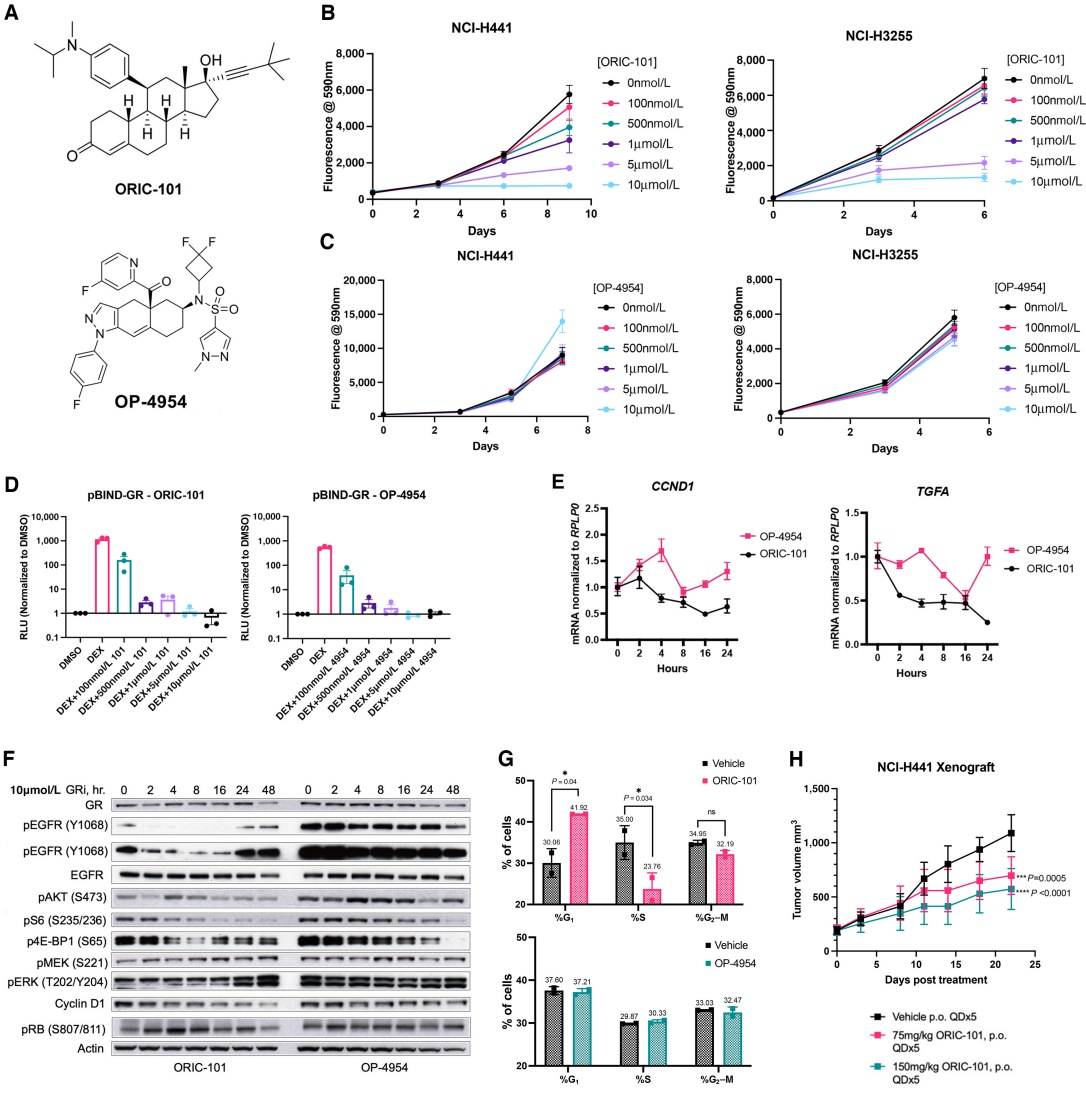
Growth promoting functions of GR in FOXA1/GR-dependent NSCLC can be inhibited by ORIC-101. **A,** Chemical structures of steroidal GR antagonist, ORIC-101 and non-steroidal GR antagonist, OP-4954. Proliferation of H441 and H3255 cells cultured in normal media and treated with indicated concentrations of ORIC-101 (**B**) or OP-4954 (**C**). Datapoints are Resazurin fluorescence normalized to DMSO treated cells, means ± SD, *n* = 6. **D,** Measurement of luciferase activity driven by glucocorticoid response element promoter in 293T cells cultured in charcoal-stripped media. Cells were treated with vehicle, 100 nmol/L dexamethasone, or 100 nmol/L dexamethasone plus indicated concentrations of ORIC-101 or OP-4954 for 24 hours. Data are shown as means ± SD of three biologically independent replicates. **E,** mRNA levels over a 24-hour period of indicated FOXA1/GR targets in H441 cells cultured in normal serum-containing media and treated with 10 μmol/L of ORIC-101 (black) or OP-4954 (pink). Values represent fold change in expression relative to 0 hour. (no treatment) condition from three replicates ± SEM. **F,** Immunoblots of FOXA1/GR targets and signaling pathways downstream of targets in H441 cells treated with 10 μmol/L of ORIC-101 or OP-4954. **G,** Cell-cycle distribution of H441 cells treated for 24 hours with DMSO or 10 μmol/L ORIC-101 or DMSO or 10 μmol/L OP-4954 as measured by DNA content using FACS, plotted as % cells in G_1_, S, and G_2_–M phases. Data are means ± SD of three independent biological repeats. *P* value calculated by two-tailed Student *t* test. **H,** Growth of H441 xenograft tumors treated with vehicle, 75 or 150 mg/kg daily. *N* = 8 mice/group, data are means ± SEM. *P* value calculated by two-tailed Student *t* test.

While ligand stimulation was not able to enhance the proliferation of FOXA1-dependent NSCLC, we and others have previously observed direct antagonism of nuclear receptors can achieve additional biologic effects beyond merely “reversal of agonism” due to the resulting changes in receptor conformation (46). We therefore investigated the effects of two structurally distinct GR antagonists produced by ORIC Pharmaceuticals: ORIC-101, a steroidal antagonist (47), and OP-4954, a nonsteroidal antagonist (Fig. 4A) in the FOXA1/GR-dependent NCI-H441 and NCI-H3255 models. ORIC-101 strongly inhibited cell proliferation in a dose-dependent manner in the two lines (99% and 85% inhibition ± SEM, respectively at 10 μmol/L) whereas the same cells were resistant to OP-4954 (50% growth induction and 20% growth inhibition ± SEM respectively at 10 μmol/L; Fig. 4B and C). To ensure that the antiproliferative effects of ORIC-101 were specifically mediated by GR inhibition, we tested ORIC-101 in GR-negative HT-29 colon cancer cells and FOXA1-independent A549 lung cancer cells. HT-29 and A549 cells were resistant to ORIC-101 (6% growth induction ± SEM and 1% growth induction ± SEM, respectively; Supplementary Fig. S3A and S3B).

Despite the opposing effects of these compounds on cell proliferation in FOXA1-dependent lines, both ORIC-101 and OP-4954 potently neutralized dexamethasone-stimulated GR transactivation. We introduced a vector containing the GAL4-activation sequence upstream of a firefly luciferase reporter into 293T cells expressing the GR ligand-binding domain fused to the GAL4 DNA-binding domain. Treatment of the 293T cells with dexamethasone induced expression of the reporter, measured by luciferase activity, which was completely ablated upon cotreatment with 5 μmol/L of either ORIC-101 or OP-4954, restoring reporter activity to unstimulated levels (Fig. 4D). The equal potency with which ORIC-101 and OP-4954 block GR transactivation provide further validation that the antiproliferative effects elicited by ORIC-101 treatment cannot be simply explained by reversal of GR agonism.

To further elucidate the basis for differences between the effects of ORIC-101 and OP-4954, we examined the effects of the compounds on GR subcellular localization, recruitment to target genes, and expression of downstream targets. Both ORIC-101 and OP-4954 drive GR nuclear localization (Supplementary Fig. S3C) and recruitment to GR target loci (Supplementary Fig. S3D) but had distinct effects on GR target expression. We assessed the expression of *TGFA* and *CCND1* transcripts over the course of treatment with 10 μmol/L of either ORIC-101 or OP-4954 and found that ORIC-101 treatment resulted in stable kinetic inhibition of these targets to sub-baseline levels. In contrast, treatment with OP-4954 led to fluctuations in the expression of these targets, which returned to baseline levels after 24 hours of treatment (Fig. 4E). Because inhibition of GR suppresses TGFA mRNA expression, we posited that this effect would result in reduced activation of TGFα’s cognate receptor EGFR. Indeed, baseline phospho-EGFR levels decreased following treatment with ORIC-101, but not OP-4954. In addition, we observed suppression of cyclin D1 protein levels in ORIC-101–treated cells, but not OP-4954–treated cells also mirroring the effect at the transcript level (Fig. 4F).

On the basis of these effects on CCND1, we examined the cell-cycle distribution of NCI-H441 cells treated with vehicle, ORIC-101, or OP-4954 using FACS. The percentage of cells in the S-phase was unaffected by OP-4954 treatment compared with vehicle treatment. In contrast, we observed that treatment with ORIC-101 resulted in a 12% reduction in the proportion of cells in the S-phase compared with vehicle treatment (Fig. 4G). The reduction in cells in the S-phase was accompanied by an accumulation of cells in the G_1_-phase (increase from 30% to 42%). In accordance, we observed reduced levels of phosphorylated Rb and an induction of PARP cleavage in cells treated with ORIC-101 but not OP-4954 (Supplementary Fig. S3E). Finally, to assess whether these antitumor effects could be achieved at doses tolerable to normal tissues we implanted NCI-H441 xenografts and treated them by oral gavage with vehicle, 75 or 150 mg/kg ORIC-101 on a 5-day-on, 2-day-off schedule for 3 weeks. Treatment with ORIC-101 reduced tumor levels of phospho-EGFR and cyclin D1 (Supplementary Fig. S3F) and suppressed tumor growth in a dose-dependent manner, with 150 mg/kg resulting in a median growth inhibition of 50% (Fig. 4H). Animals treated with these doses experienced a 6% weight loss compared with 3% weight gain in vehicle-treated mice (Supplementary Fig. S3G). In contrast, treatment with ORIC-101 in the H3255 and H2009 xenograft models did not promote strong tumor growth inhibition (data not shown). Together, these data demonstrate that GR antagonism can suppress key proproliferative targets such as CCND1 and TGFα leading to antitumor effects in a subset of FOXA1-dependent NSCLC.

## Discussion

In this study, we identified and characterized a subset of NSCLC that are dependent on the expression of the pioneer TF FOXA1. Previous studies have implicated FOXA1 as a vulnerability factor in models of LUAD (48, 49), but its precise oncogenic functions in this context have not been defined. Through proteomic analysis of chromatin bound FOXA1 complexes, our study identifies an interaction with the nuclear receptor, GR, that facilitates expression of several proproliferative genes such as CCND1 and TGFA. This growth program appears to be targetable with pharmacologic antagonism of GR with ORIC-101 showing antitumor effects both *in vitro* and *in vivo*, pointing to the potential for GR antagonism as a testable therapeutic strategy.

Recent work identifying somatic alterations in *FOXA1* in cancer have further revealed the myriad of functions this pioneer factor can play in promoting tumor growth (50–52). Several groups have established the importance of FOXA1 in NSCLC based in part on its interactions with NKX2 (48, 53–55). We speculated that FOXA1 might also interact with proproliferative TFs given the broad pattern of dependency that is observed in models harboring a variety of driver alterations such as KRAS or EGFR. To uncover such interactions, we leveraged the screening power of RIME that can potentially identify even weak or transient protein–protein interactions (23, 28). Using this approach, the top scoring hit among TFs was GR which has been previously associated with therapeutic resistance (38, 56), and cell survival (39, 57) in lung cancer. While FOXA1/GR dependence could be observed in a subset of NSCLC, this was not uniformly the case despite ubiquitous expression of FOXA1 and GR across cell lines. This has strong analogies to the situation with ER in FOXA1-dependent breast cancer where only a subset of ER-expressing tumors depends on ER for survival. However, the potential for GR to promote growth in even a small subset of NSCLC was intriguing and so we sought to understand the basis for this dependency.

By analyzing GR and FOXA1 cistromes and transcriptomes, we found that FOXA1 and GR colocalized on canonical proproliferative genes, CCND1 and TGFA. Whereas GR binding at these target loci was not detectable absent ligand stimulation, expression of these transcripts was unaffected by dexamethasone treatment. Given these findings, we cannot exclude the possibility that GR regulation of proproliferative gene targets require alternative mechanisms of activation (e.g., posttranslational modification) or is nongenomic in nature. Nonetheless, the observation that knockdown of FOXA1 or GR leads to target suppression and growth inhibition combined with the potential for pharmacologic targeting the ligand-binding domain of GR establishes the notion of targeting GR as a therapeutic strategy.

In line with our observation that FOXA1 and GR regulate target gene expression and proliferation in an agonist-independent manner, we found that compounds designed to antagonize GR solely via competition with an agonist to reverse receptor transactivation, such as OP-4954, were ineffective in suppressing the GR growth program. In contrast, compounds that can inhibit both ligand-dependent and ligand-independent functions of GR, such as ORIC-101, suppress the expression of FOXA1/GR targets below baseline levels, and inhibit proliferation and tumor growth in a dose-dependent manner. These findings should lay the foundation for future mechanistic studies to elucidate the specific structures and TF complexes induced by these distinctive inhibitors.

While our finding of a FOXA1-GR dependency for a subset of NSCLC nominates an intriguing new therapeutic approach for this disease, it is unlikely that GR-targeted therapy will be efficacious as a single agent in a tumor type where nearly 60% of patients harbor genomic alterations hyperactivating RAS and EGFR. Therefore, additional work is needed to both generate biomarkers that identify this dependent subset as well as test rational combinations of therapy that can make this approach more clinically impactful. Interestingly, the potential to coordinately downregulate both upstream and downstream components of the EGF pathway raises the possibility that this may combine well with drugs targeting the mutated components of these pathways such as EGFR or KRAS and serve as a method to simultaneously combat multiple feedback adaptations that challenge single-agent oncoprotein therapies.

## Supplementary Material

Supplemental Table 1 FOXA1 gene effect scores in NSCLC cell lines from DepMap

Supplemental Table 2 Identification of proteins in FOXA1 and GR interactomes by RIME

Supplemental Table 3 Shared target genes of FOXA1 and GR in NCI-H441 cells

Supplementary Figure S1 Supplemental figure S1 data panels and legend accompanying main body figure 2. Supplementary Figure S1 shows GR is the top-ranked interacting protein of FOXA1 in FOXA1-dependent NSCLC cell lines models. Additionally, FOXA1-dependent, but not FOXA1-independent NSCLC cells depend on GR for sustained proliferation.

Supplementary Figure S2 Supplemental figure S2 data panels and legend accompanying main body figure 3. Supplementary figure S2 shows that dexamethasone treatment does not enhance proliferation of FOXA1/GR -dependent NSCLC, and FOXA1/GR target gene expression is not driven by ligand activation of GR.

Supplementary Figure S3 Supplemental figure S3 data panels and legend accompanying main body figure 4. Supplementary figure S3 demonstrates that ORIC-101 does not inhibit the proliferation of GR independent and GR- NSCLC models. ORIC-101 treatment leads to GR nuclear translocation, binding to FOXA1-GR target genes, and induction of apoptosis. Finally, ORIC-101 is efficacious at doses tolerable to mice and leads to inhibition of FOXA1-GR targets in vivo.
