# Supplementary material for: A Druggable FOXA1-Glucocorticoid Receptor Transcriptional Axis Drives Tumor Growth in a Subset of Non-Small Cell Lung Cancer
Source: Cancer Res Commun. 2023 Sep 7;3(9):1788–99. doi: 10.1158/2767-9764.CRC-23-0310 (PMC10484118; doi:10.1158/2767-9764.CRC-23-0310)
Supplement: Supplementary Figure S1 — Supplemental figure S1 data panels and legend accompanying main body figure 2. Supplementary Figure S1 shows GR is the top-ranked interacting protein of FOXA1 in FOXA1-dependent NSCLC cell lines models. Additionally, FOXA1-dependent, but not FOXA1-independent NSCLC cells depend on GR for sustained proliferation. [file crc-23-0310-s04.pdf]

Figure S1

A

Protein	H441 # peptides	H3255 # peptides	HCC44 # peptides	H2009 # peptides	H1373 # peptides	Mascot Ion score
FOXA1	8	0	7	2	3	121
GR	5	3	1	0	0	121
BCL6	4	1	0	1	0	118
STAT3	2	0	8	1	0	100.2
JUNB	1	1	3	1	0	82.5
RUNX1	4	2	3	1	0	80.7
JUN	0	1	1	2	0	79.9

B

Protein	H441 # peptides	H3255 # peptides	HCC44 # peptides	H2009 # peptides	H1373 # peptides	Mascot Ion score
ARID1A	11	0	35	10	2	122.2
SMARCE1	1	1	11	3	0	110.7
KMT2C	16	17	22	29	5	110.3
SMARCA4	1	0	38	4	0	106.6
NCOA6	11	5	13	15	0	104.8
HDAC1	3	1	12	1	0	98.2
KDM6A	5	3	14	12	0	94.3
ASH2L	1	0	7	2	0	83.9
KDM1A	2	0	6	2	0	82.8
DPY-30	0	2	3	2	0	80.7
HDAC3	7	1	4	1	1	79.3
NCOR1	7	0	23	4	1	78.5
NCOR2	10	0	8	2	0	63
BAG3	1	3	1	2	0	60.8

C

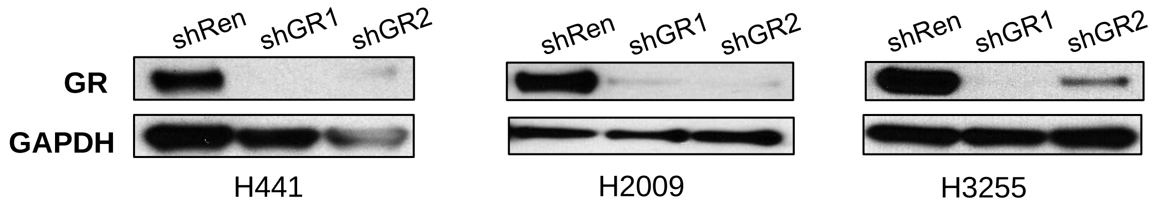

D

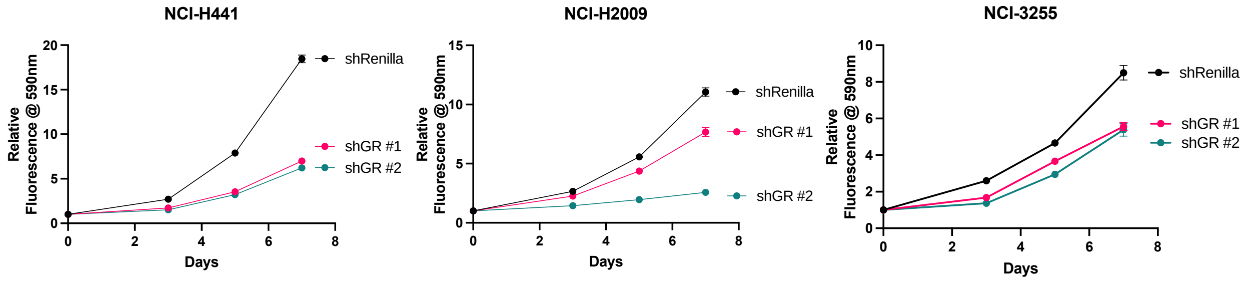

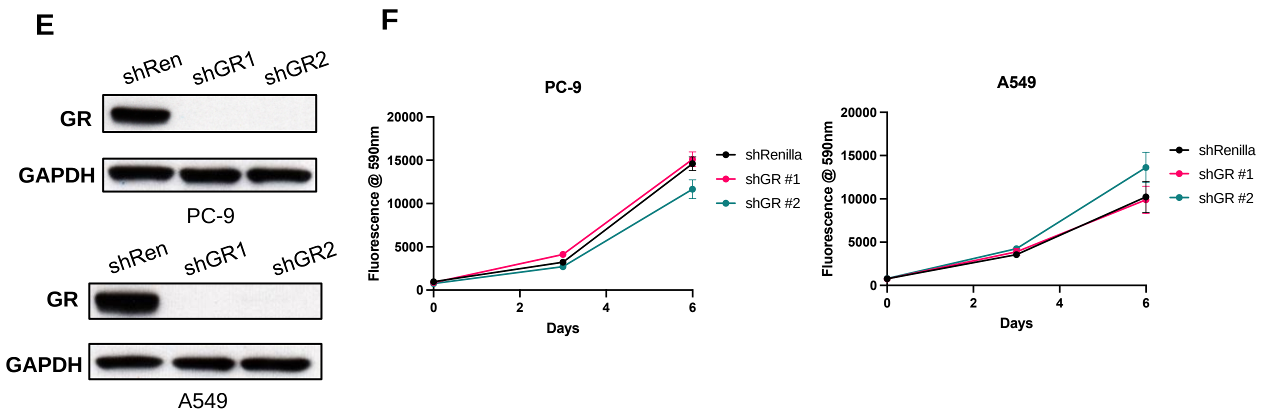

Supplemental Figure S1. FOXA1 interacts with GR in FOXA1-dependent NSCLC. A) Table depicting the number of unique peptides identified and the Mascot ion score for TFs present in the FOXA1 interactome in FOXA1-dependent NSCLC cell lines. **B)** Table depicting the number of unique peptides identified and Mascot ion score for co-regulators and chromatin remodelers present in the FOXA1 interactome in FOXA1-dependent NSCLC cell lines. **C, E)** Immunoblot of GR and GAPDH protein levels in Renilla and GR-knockdown isogenic cell lines. **D, F)** Effect of shRNA knockdown on NSCLC cell proliferation. Data points represent the resazurin fluorescence (± SD, n=6).
