# Supplementary material for: A Druggable FOXA1-Glucocorticoid Receptor Transcriptional Axis Drives Tumor Growth in a Subset of Non-Small Cell Lung Cancer
Source: Cancer Res Commun. 2023 Sep 7;3(9):1788–99. doi: 10.1158/2767-9764.CRC-23-0310 (PMC10484118; doi:10.1158/2767-9764.CRC-23-0310)
Supplement: Supplementary Figure S2 — Supplemental figure S2 data panels and legend accompanying main body figure 3. Supplementary figure S2 shows that dexamethasone treatment does not enhance proliferation of FOXA1/GR -dependent NSCLC, and FOXA1/GR target gene expression is not driven by ligand activation of GR. [file crc-23-0310-s05.pdf]

Figure S2

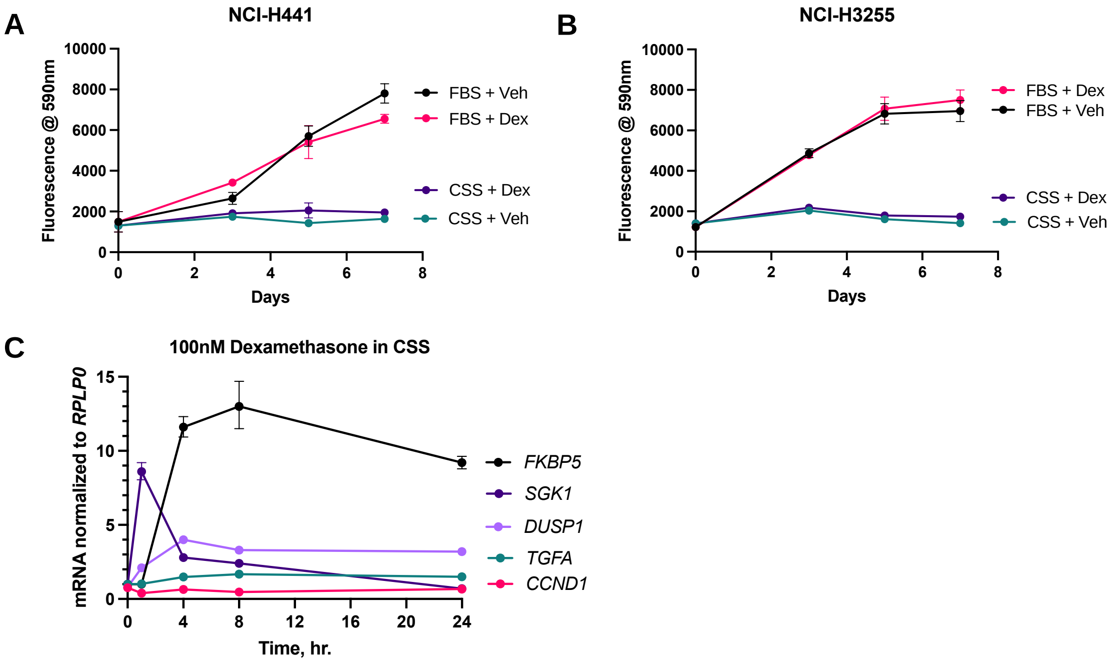

Supplemental Figure S2. Growth promoting functions of GR are uncoupled from corticosteroid activated-functions of GR in FOXA1/GR-dependent NSCLC. Proliferation of **A)** H441 cells or **B)** H3255 cells cultured in full serum-containing media or charcoal-stripped serum-containing media and treated with either vehicle or 100nM dexamethasone on day 0. Data points are resazurin fluorescence at 590 nm, mean ± SD, n=6. **C)** H441 cells were cultured in charcoal-stripped media and treated with 100nM dexamethasone. Expression of FOXA1/GR targets was assayed at the indicated time points by qPCR (mean ± SD, n=3). The mRNA levels were normalized to the CT values in full serum media.
