# Supplementary material for: A Druggable FOXA1-Glucocorticoid Receptor Transcriptional Axis Drives Tumor Growth in a Subset of Non-Small Cell Lung Cancer
Source: Cancer Res Commun. 2023 Sep 7;3(9):1788–99. doi: 10.1158/2767-9764.CRC-23-0310 (PMC10484118; doi:10.1158/2767-9764.CRC-23-0310)
Supplement: Supplementary Figure S3 — Supplemental figure S3 data panels and legend accompanying main body figure 4. Supplementary figure S3 demonstrates that ORIC-101 does not inhibit the proliferation of GR independent and GR- NSCLC models. ORIC-101 treatment leads to GR nuclear translocation, binding to FOXA1-GR target genes, and induction of apoptosis. Finally, ORIC-101 is efficacious at doses tolerable to mice and leads to inhibition of FOXA1-GR targets in vivo. [file crc-23-0310-s06.pdf]

Figure S3

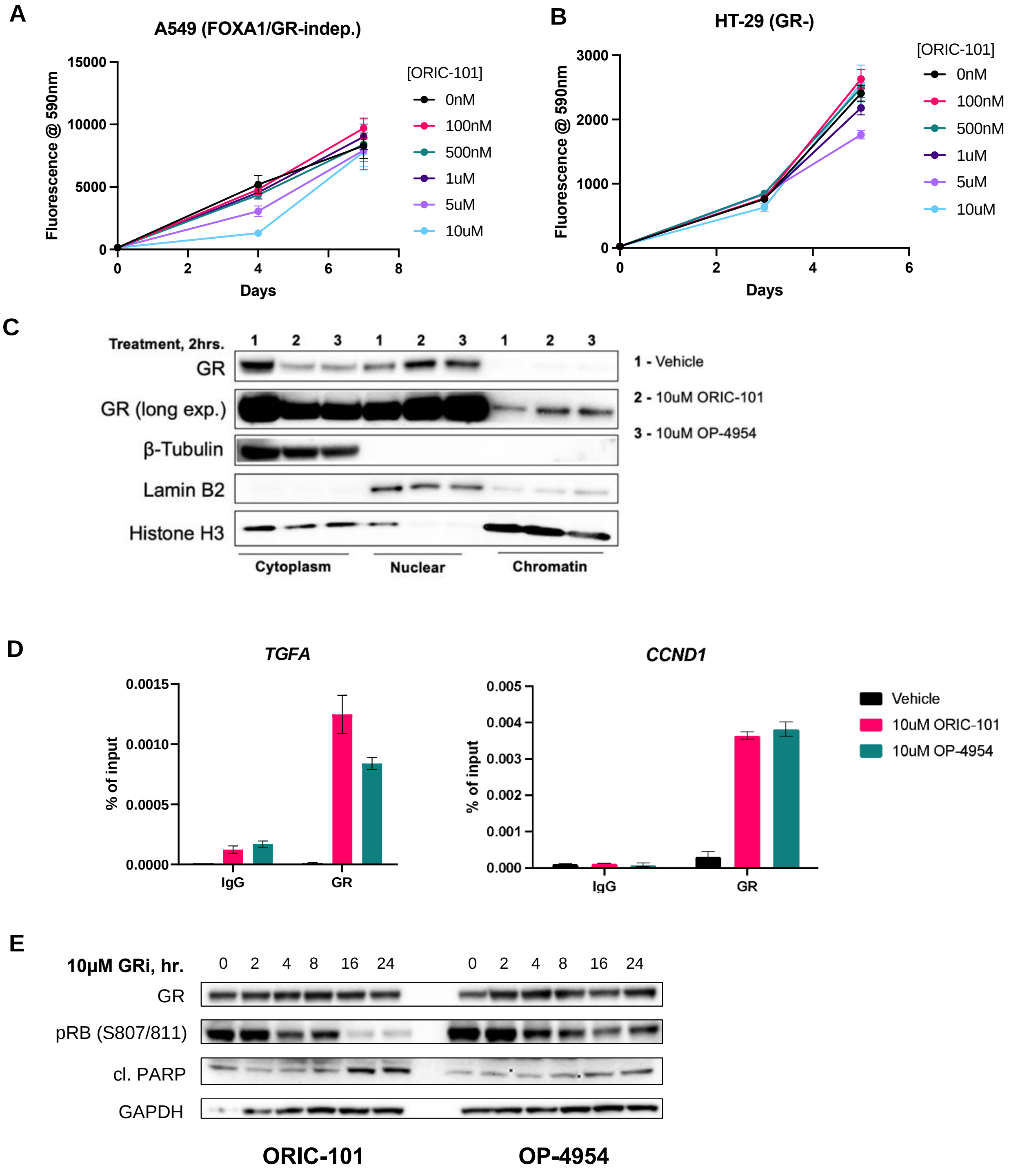

A
A549 (FOXA1/GR-indep.)
Fluorescence @ 590nm
15000
10000
5000
0
0
2
4
6
8
Days
[ORIC-101]
0nM
100nM
500nM
1uM
5uM
10uM
B
HT-29 (GR-)
Fluorescence @ 590nm
3000
2000
1000
0
0
2
4
6
Days
[ORIC-101]
0nM
100nM
500nM
1uM
5uM
10uM
C
Treatment, 2hrs.
1 2 3 1 2 3 1 2 3
GR
GR (long exp.)
β-Tubulin
Lamin B2
Histone H3
Cytoplasm
Nuclear
Chromatin
1 - Vehicle
2 - 10uM ORIC-101
3 - 10uM OP-4954
D
TGFA
% of input
0.0015
0.0010
0.0005
0.0000
IgG
GR
CCND1
% of input
0.005
0.004
0.003
0.002
0.001
0.000
IgG
GR
Vehicle
10uM ORIC-101
10uM OP-4954
E
10μM GRi, hr.
0 2 4 8 16 24
0 2 4 8 16 24
GR
pRB (S807/811)
cl. PARP
GAPDH
ORIC-101
OP-4954

F

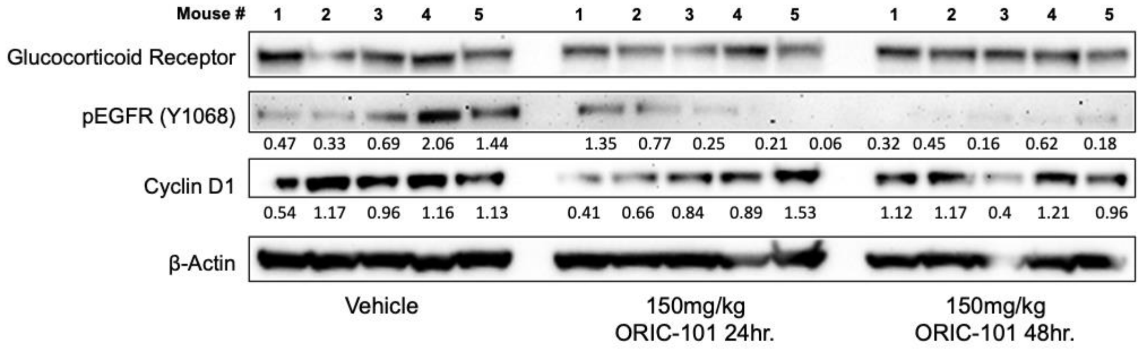

G

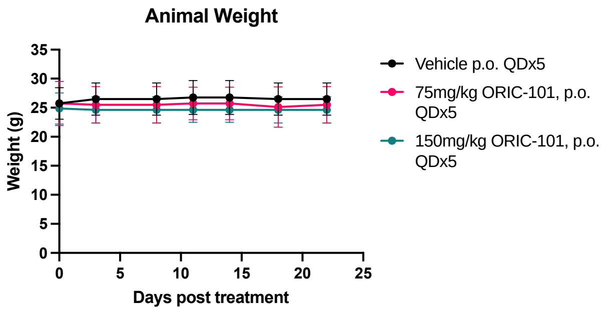

Supplemental Figure S3. Growth promoting functions of GR in FOXA1/GR-dependent NSCLC can be inhibited by ORIC-101. A) Proliferation of A549 and **B)** HT-29 cells treated with the indicated concentrations of ORIC-101. Data points are resazurin fluorescence at 590 nm, mean ± SD, n=6. **C)** Immunoblots of fractionated H441 cells after 2hr. treatment with DMSO, ORIC-101 or OP-4954. Comparisons can be made within fractions, but not across fractions. **D)** ChIP-qPCR analysis of GR, following 2hrs. of indicated treatment. Rabbit isotype IgG was used as a negative control. Values correspond to mean percentage of input chromatin +/- SEM of duplicate qPCR reactions. **E)** Immunoblots of the indicated proteins in H441 treated with 10uM ORIC-101 or 10uM OP-4954. **F)** Immunoblots of tumors from NCI-H441 xenografted mice treated with vehicle or 150 mg/kg ORIC-101 for 24 or 48 h. Western blot bands were quantified using Image J, and relative intensity was calculated by dividing the signal intensity of the individual band by the average signal intensity of the vehicle treated tumors. **G)** Weights of NCI-H441 xenografted mice treated with vehicle, 75mg/kg or 150mg/kg daily. N = 8 mice/group. Data are mean weights +/- SD.
